# Risk factors and prognostic value of endotoxemia in patients with acute myocardial infarction

**DOI:** 10.3389/fcvm.2024.1419001

**Published:** 2024-06-25

**Authors:** Maxime Nguyen, Alain Putot, David Masson, Yves Cottin, Thomas Gautier, Laura Tribouillard, Anne-Laure Rérole, Pierre-Grégoire Guinot, Maud Maza, Jean-Paul Pais de Barros, Valérie Deckert, Michel Farnier, Laurent Lagrost, Marianne Zeller

**Affiliations:** ^1^ Department of Anesthesiology and Intensive Care, Dijon University Hospital, Dijon, France; ^2^ Lipides Nutrition Cancer UMR1231 and LipSTIC LabEx, Université de Bourgogne, Dijon, France; ^3^ Geriatrics Internal Medicine Department, Dijon University Hospital, Dijon, France; ^4^ Physiopathologie et Epidémiologie Cérébro-Cardiovasculaires (PEC2), EA7460, Université de Bourgogne Franche-Comté, Dijon, France; ^5^ Infectious Diseases and Internal Medicine Department, Hôpitaux du Pays du Mont Blanc, Sallanches, France; ^6^ Cardiology Department, Dijon University Hospital, Dijon, France

**Keywords:** endotoxemia, lipopolysaccharide, acute myocardial infarction, inflammation, diabetes, lipoprotein

## Abstract

**Background:**

There is increasing evidence regarding the association between endotoxemia and the pathogenesis of atherosclerosis and myocardial infarction (MI). During the acute phase of MI, endotoxemia might increase inflammation and drive adverse cardiovascular (CV) outcomes. We aimed to explore the risk factors and prognostic value of endotoxemia in patients admitted for acute MI.

**Methods:**

Patients admitted to the coronary care unit of Dijon University Hospital for type 1 acute MI between 2013 and 2015 were included. Endotoxemia, assessed by plasma lipopolysaccharide (LPS) concentration, was measured by mass spectrometry. Major adverse CV events were recorded in the year following hospital admission.

**Results:**

Data from 245 consecutive MI patients were analyzed. LPS concentration at admission markedly increased with age and diabetes. High LPS concentration was correlated with metabolic biomarkers (glycemia, triglyceride, and total cholesterol) but not with CV (troponin Ic peak and N-terminal pro-brain natriuretic peptide) or inflammatory biomarkers (C-reactive protein, IL6, IL8, and TNFα). LPS concentration was not associated with in-hospital or 1-year outcomes.

**Conclusions:**

In patients admitted for MI, higher levels of endotoxins were related to pre-existing conditions rather than acute clinical severity. Therefore, endotoxins measured on the day of MI could reflect metabolic chronic endotoxemia rather than MI-related acute gut translocation.

## Introduction

Myocardial infarction (MI) is a frequent event that carries high morbidity and mortality worldwide. Bacterial endotoxins (lipopolysaccharides, LPSs) are pathogen-associated molecular patterns (PAMPs) that are part of the outer membrane of Gram-negative bacteria. LPSs have been described as exerting a noxious effect by triggering inflammation (TLR-4 recognition and NF-kB transcription) (1).

There is increasing evidence that endotoxemia is associated with MI through multiple underlying mechanisms. First, chronic low-grade endotoxemia has been reported in metabolic disorders (2, 3), defining the concept of metabolic endotoxemia (4). In addition, LPSs are closely linked to lipoprotein metabolism by reverse LPS transport (5, 6). These conditions are risk factors for MI, and emerging concepts suggest that endotoxemia has a role in coronary artery disease (CAD) and the genesis of MI (7, 8). Second, several studies have suggested an alteration of the gut barrier function at the acute phase of MI, resulting in acute endotoxemia (9, 10). Because endotoxins reduce cardiac performance (11) and cause vasoplegia (12), they might increase the risk of heart failure after MI and worsen short-term patient outcomes. Finally, inflammation is thought to extend myocardial injury (13) and promote recurrent cardiovascular (CV) events (14), so endotoxemia might also have negative effects on long-term recovery. Despite these potentially harmful effects, data regarding acute endotoxemia and MI are scarce and further evidence is needed (15).

The primary objective of the present study was to explore factors associated with endotoxemia at hospital admission in patients with MI and to determine whether endotoxemia at hospital admission was related to metabolic conditions (thus chronic low-grade translocation promoting atherosclerosis) or to acute gut barrier failure in the context of MI. The secondary objective was to determine the association of endotoxemia with inflammation, initial MI severity, and short-term and long-term outcomes.

## Methods

### Patients

This study is an ancillary analysis of a prospectively acquired database (RICO survey) (16). All consecutive patients admitted to the coronary intensive care unit (ICU) of the Dijon University Hospital (France) from January 2013 to April 2015 for type 1 MI were prospectively included. Patients with prior coronary arterial disease [MI, transluminal angioplasty, unstable angina, or coronary artery bypass graft (CABG) surgery] or chronic kidney disease were excluded. The present study agrees with the ethical guidelines of the Declaration of Helsinki. Informed consent was obtained from the participants before their inclusion in the study, and the Ethics Committee of the University Hospital of Dijon approved the protocol (BIOCARDIS-2016–9205AAO034S02117).

### Data collection

Patient characteristics, including age and gender, were obtained at hospital admission, along with medical history (hypertension, diabetes, smoking, and family history of coronary artery disease, main treatments [antiplatelets, angiotensin 2 receptor blockers (ARB), angiotensin 2 converting enzyme (ACE) inhibitors, statins, and beta-blockers], and clinical data [left ventricular ejection fraction, heart rate (HR), blood pressure, catecholamine administration, and infarction location]. Shock index was defined as the heart rate divided by systolic blood pressure (SBP). Coronary artery disease burden at coronary angiography through multivessel disease and the SYNTAX (17) score were also collected. The Global Registry of Acute Coronary Events (GRACE) risk score (18), a robust prognosis tool following MI, was also calculated.

### Determination of blood markers

Blood samples were taken on admission. N-terminal pro-brain natriuretic peptide (Nt-ProBNP, normal value <125 pg/ml), blood lipids [normal range 1.20–2.4 g/L for cholesterol, 0.4–0.6 g/L for HDL cholesterol, and 0.4–1.5 g/L for triglycerides (TG)], glucose (normal range 4.3–6.4 mmol/L), glycated hemoglobin (HbA1c, normal value <6%), and C-reactive protein (CRP) (normal value <4 mg/L) were measured. The troponin Ic peak (normal value <0.1 µg/L) was obtained from three blood samples within 24 h following admission. The estimated glomerular filtration rate (eGFR) was calculated using the chronic kidney disease-epidemiology collaboration formula (CKD-EPI).

Plasma was taken upon ICU admission in an EDTA (ethylenediaminetetraacetic acid) blood collection tube. Blood was centrifugated (2,000×*g* for 10 min at 4 °C), and plasma was stored at −80 °C. LPS, lipid transfer protein activities [phospholipid transfer protein (PLTP) and cholesteryl ester transfer protein (CETP)], and cytokines were retrospectively measured from this plasma collection.

LPS was quantified by measuring one of its components [3-hydroxy myristate (3HM)] using liquid chromatography coupled with tandem mass spectrometry, as previously described (19). Briefly, samples were hydrolyzed for 3 h at 90 °C in the presence of hydrochloric acid to release free fatty acids, which were subsequently extracted with water and ethane/ethyl acetate (3/2 v/v). Dried extracts were dissolved in ethanol and injected onto an SBC18 2.1 mm × 50 mm, 1.8 µm column, connected to an Infinity 1290 HPLC system (Agilent Technologies). Separation of hydroxylated fatty acids was achieved at 45 °C at a flow rate of 0.4 ml/min using ammonium formate (5 mM/0.1% formic acid) as eluent A (55% for 0.5 min) and 95% acetonitrile as eluent B (100% reached in 2.5 min and maintained for 5 min). MS/MS detection was performed in negative mode using a QqQ 6490 triple quadrupole mass spectrometer equipped with a JetStream ESI source to quantify the selected ions as follows: for 3HM, precursor ion 243.2 Da and product ion 59 Da; for 3-hydroxytridecanoic acid (IS), which was used as the internal standard, precursor ion 229.2 Da and product ion 59 Da.

PLTP and CETP activities were measured in undiluted plasma using commercially available fluorescence activity assays (Roar Biomedical), according to the manufacturer's instructions. Incubations were performed at 37 °C for 30 min (for PLTP) or 3 h (for CETP), with fluorescence monitoring (excitation, 465 nm; emission, 535 nm) throughout the incubation period with a Victor2 multilabel counter (PerkinElmer, Waltham, United States). Transfer activities were calculated from the slope of fluorescence increase between 1 and 30 min (for PLTP) or between 1 min and 1 h (for CETP) and expressed as arbitrary fluorescence units (AU).

Cytokines were measured using a Luminex® Human Magnetic assay (R&D Systems, Minneapolis, USA). The assays were performed according to the manufacturer's instructions (including for sample collection and preparation). Plasma was not diluted for this analysis. Standards and samples were analyzed on a Luminex® apparatus (BioPlex 200, Bio-Rad, Munich, Germany) using BioPlex Manager Software (Version 5, Bio-Rad, Hercules, CA, USA). For all cytokines, standard curves ranged from 3.2 to 10,000 pg/ml.

### Outcomes

Plasma markers included endotoxin plasma concentration, cytokine plasma concentration, and CRP.

Clinical outcomes included in-hospital major adverse CV events (MACEs), such as CV death, heart failure (defined as Killip class >1), and recurrent myocardial infarction, ventricular and supraventricular arrhythmia, and ICU length of stay. A follow-up at 1 year with the patient, next of kin, or the treating physician was conducted by mail or telephone. Occurrences of MACEs, defined as CV death, recurrent myocardial infarction, hospitalization for heart failure, unscheduled percutaneous coronary intervention (PCI), coronary artery bypass surgery, unstable angina or angina pectoris, were collected.

### Statistical analysis

No sample size calculation was performed for this study. All the patients included in the cohort were analyzed. Dichotomous variables are expressed as *n* (%), and continuous variables are expressed as means ± standard deviations or medians [interquartile ranges (IQR)]. A Kolmogorov–Smirnov test was performed to assess the normality of continuous variables. Non-normally distributed variables were log-transformed when needed. The Mann–Whitney test or Student's *t*-test was used to compare continuous data, as appropriate. Pearson correlation analyses (for normally distributed variables) or Spearman correlation analyses (one or two non-Gaussian variables) were performed. The threshold for significance was set at *p* < 0.05. Multivariate linear regression models were built to estimate LPS levels based on significant variables in univariate analysis, with an inclusion threshold of *p* < 0.10. Variable selection was stepwise. The normality of residuals and homoscedasticity were checked graphically; the model was also checked for autocorrelations of residuals (Durbin–Watson test) and multicollinearity (variance inflation factor). Missing data were considered at random and were omitted. SPSS version 12.0.1 (IBM Inc., Armonk, NY, USA) was used for all analyses.

## Results

### Baseline characteristics

Two hundred and forty-five patients were analyzed. Baseline characteristics are presented in Table 1. The mean age was 62 ± 13 years, most patients were men (72%), and the median body mass index (BMI) was 27 (25–29). We identified 92 patients (37.6%) with hypercholesterolemia and 34 (14%) with diabetes. Most patients had ST-segment elevation (Table 2).

**Table 1:** Association between LPS plasma concentration and patient's characteristics.

Variable		*n* (%), mean ± SD or median (IQR) *N* = 245	Total LPS or *r*	*p*
Demographic characteristics				
Age, years		62 ± 13	0.173	0.007
Female	No	176 (71.8%)	106.36 (82.45–131.16)	0.946
	Yes	69 (28.2%)	106.18 (84.47–129.99)	
BMI, kg/m^2^		27 (25–29)	0.192	0.003
Medical history				
Hypertension	No	139 (56.7%)	104.78 (83.16–127.42)	0.521
	Yes	106 (43.3%)	108.23 (86.14–133.28)	
Diabetes	No	211 (86.1%)	103.03 (82.45–124.74)	<0.001
	Yes	34 (13.9%)	139.60 (113.86–230.62)	
Family history of CAD	No	168 (68.6%)	108.98 (83.49–133.13)	0.141
	Yes	77 (31.4%)	102.95 (83.11–125.39)	
Current smoking	No	137 (55.9%)	110.44 (91.60–132.65)	0.022
	Yes	108 (44.1%)	102.06 (79.58–121.80)	
Chronic treatments				
Antiplatelet	No	241 (98.4%)	106.32 (83.30–131.00)	0.938
	Yes	4 (1.6%)	107.04 (77.49–133.89)	
Aspirin	No	223 (91.0%)	106.13 (83.44–129.14)	0.570
	Yes	22 (9.0%)	115.68 (74.40–139.42)	
ARB	No	209 (85.3%)	106.07 (82.75–128.84)	0.191
	Yes	36 (14.7%)	115.28 (95.36–136.59)	
ACE inhibitors	No	219 (89.4%)	106.32 (83.06–130.28)	0.572
	Yes	26 (10.6%)	107.20 (88.77–144.31)	
Statin	No	214 (87.3%)	107.88 (83.72–131.71)	0.173
	Yes	31 (12.7%)	98.82 (75.14–121.03)	
Beta-blockers	No	210 (85.7%)	106.36 (83.06–130.38)	0.961
	Yes	35 (14.3%)	104.61 (90.90–133.92)	

Data are expressed as *n* (%), mean ± standard deviation (SD), or median (IQR).

LPS is expressed as 3HM (pmol/L). *p*-values are given for the association between each variable and LPS plasma concentration.

**Table 2:** Association between LPS plasma concentration and clinical data on admission.

Variable		*n* (%), mean ± SD, or median (IQR) *N* = 245	Total LPS or *r*	*p*
Clinical data				
LVEF, %		55 (45–60)	−0.069	0.283
LVEF <40%	No	219 (89.8%)	106.18 (83.06–128.54)	0.203
	Yes	25 (10.2%)	113.98 (90.45–155.10)	
HR, bpm		76 (67–88)	0.191	0.003
SBP, mmHg		141 ± 27	0.131	0.043
DBP, mmHg		83 ± 19	0.100	0.123
Catecholamine	No	230 (93.9%)	106.15 (83.37–130.54)	0.827
	Yes	15 (6.1%)	113.98 (83.06–131.83)	
Shock index ≥ 0.7	No	191 (80.3%)	104.78 (82.22–125.75)	0.034
	Yes	47 (19.7%)	117.01 (90.64–163.53)	
STEMI	No	97 (39.6%)	103.87 (79.72–125.67)	0.181
	Yes	148 (60.4%)	108.99 (85.24–133.56)	
Anterior wall location	No	150 (61.2%)	106.10 (82.91–128.31)	0.396
	Yes	95 (38.8%)	110.24 (85.18–133.51)	
Heart failure	No	201 (82.0%)	108.51 (83.69–131.75)	0.162
	Yes	44 (18.0%)	96.28 (79.71–122.72)	
Time to admission, min		160 (101–338)	0.038	0.549
GRACE risk score		137 ± 36, *n* = 238	0.110	0.089
Angiographical data				
Coronary angiography	No	2 (0.8%)	94.60 (83.06-x)	0.515
	Yes	243 (99.2%)	106.40 (83.44–131.32)	
SYNTAX score		8 (5–14), *n* = 238	0.071	0.275
Multivessel disease	No	137 (55.9%)	106.18 (84.49–129.29)	0.811
	Yes	108 (44.1%)	107.33 (81.79–131.58)	
PCI	No	45 (18.4%)	105.62 (84.78–123.27)	0.641
	Yes	200 (81.6%)	108.23 (83.08–132.14)	
Plasma markers				
Creatinine, µmol/L		77 ± 15	−0.054	0.400
Troponin Ic peak, ng/ml		26.0 (5.3–93.5)	0.077	0.228
Nt-ProBNP, pg/ml		400 (103–1,247)	0.000	0.996

DBP, diastolic blood pressure; STEMI, ST-elevation myocardial infarction.

Data are expressed as *n* (%), mean ± standard deviation (SD), or median (IQR). LPS is expressed as 3HM (pmol/L). The shock index was calculated as the ratio of heart rate to systolic blood pressure. *p*-values are given for the association between each variable and LPS plasma concentration. *p*-values presented are not corrected for multiple comparisons.

### Variables associated with endotoxemia

**Figure 1:**
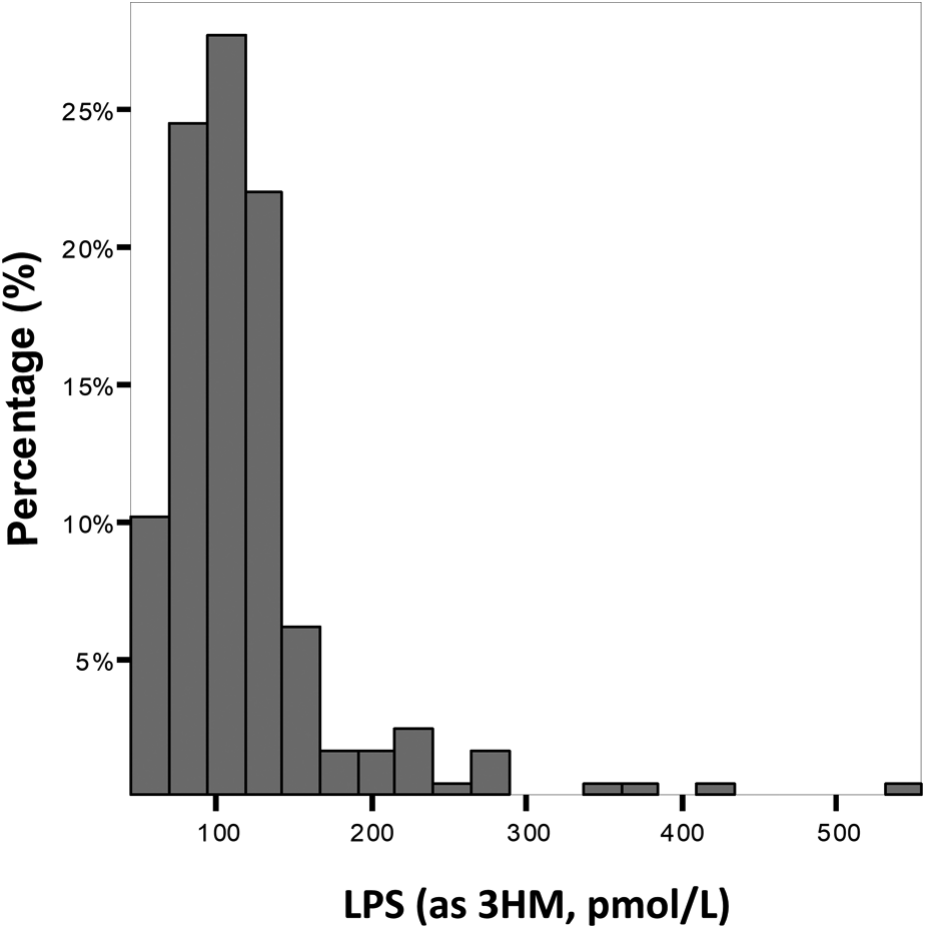
Distribution of the population according to LPS concentration at admission. Results are expressed as % of the population for each range of LPS concentration.

**Figure 2:**
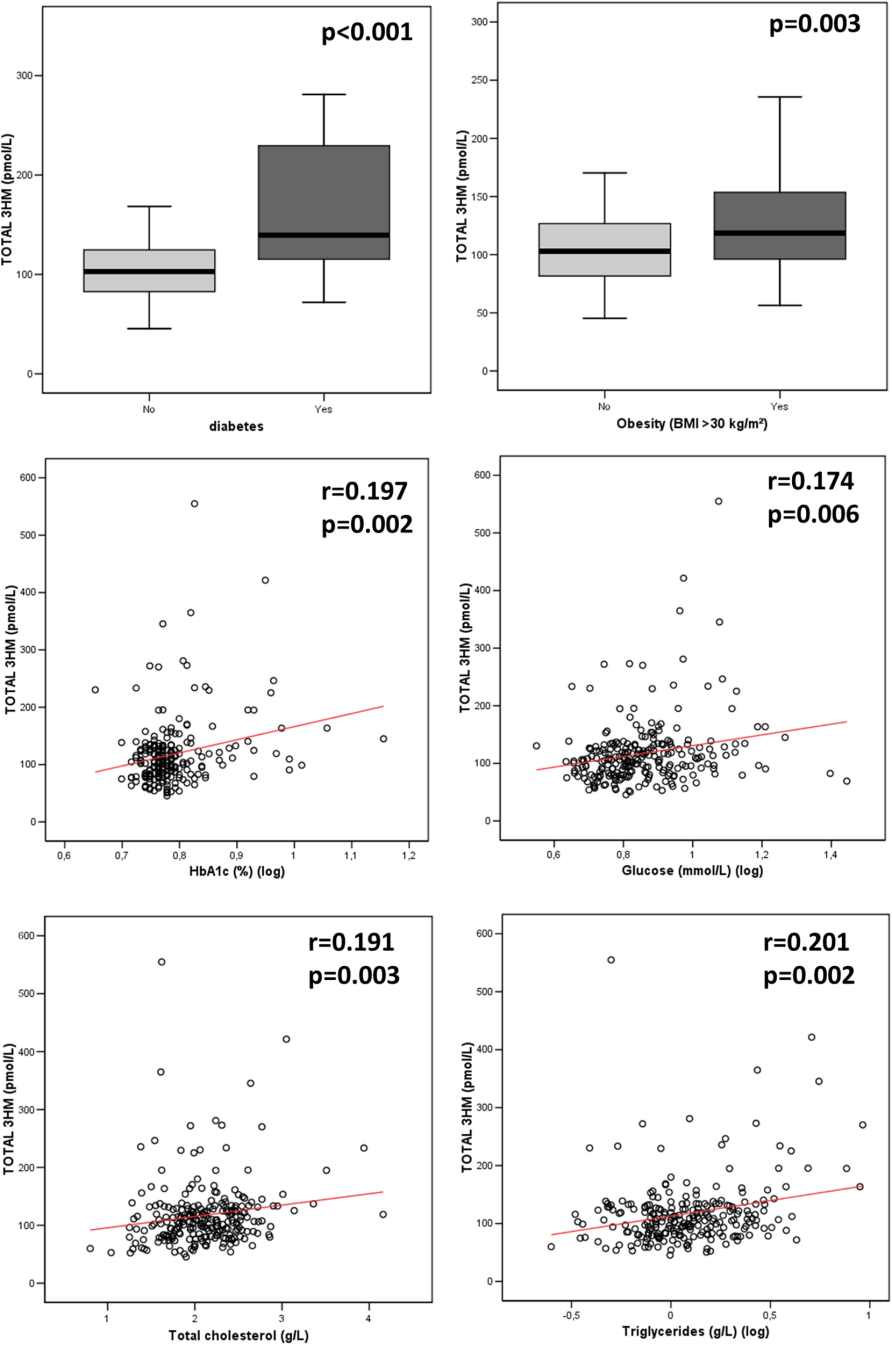
Association between LPS concentration and metabolic parameters at admission. *p*-values were computed using the Spearman test for correlation and the Mann–Whitney test for group comparisons. *n* = 235 for HbA1c, 245 for glycemia, 245 for diabetes, and 244 for total cholesterol, triglyceridemia, and obesity. 3HM, 3-hydroxy myristate.

The median LPS concentration was 106 (83–131) pmol/L of 3HM. The distribution of LPS concentration in our population is illustrated in Figure 1. Endotoxemia was associated with age, diabetes, and obesity (Table 1, Figure 2), as well as with total cholesterol (*r* = 0.191, *p* = 0.003), triglyceridemia (*r* = 0.201, *p* = 0.002), and glucose metabolism parameters (blood glucose, HbA1c) (Table 3). LPS was lower in smokers (102.06 pmol/L of 3HM (79.58–121.80) vs. 110.44 pmol/L of 3HM (91.60–132.65), *p* = 0.22). PLTP activity was positively correlated with LPS concentrations (*r* = 0.146, *p* = 0.022). At admission, patients with high endotoxemia had a faster heart rate (*r* = 0.19, *p* < 0.01), and a higher shock index (≥0.7) was associated with higher LPS concentrations. In multivariate analysis, triglycerides, total cholesterol, history of diabetes, and age were independently associated with LPS concentration (Table 4).

**Table 3:** Association between LPS plasma concentration and lipid and glucose metabolism.

Variable		*n* (%) or median (IQR) *N* = 245	Total LPS or *r*	*p*
LDL cholesterol, g/L		1.32 (1.09–1.60)	0.099	0.124
HDL cholesterol, g/L		0.47 (0.38–0.56)	0.095	0.138
Total cholesterol, g/L		2.09 (1.80–2.40)	0.191	0.003
Triglycerides, g/L		1.16 (0.81–1.80)	0.201	0.002
PLTP, AU		186.7 ± 33.0	0.146	0.022
CETP, AU		31.3 ± 11.3	−0.051	0.424
LBP, µg/ml		26.1 ± 10.2	−0.132	0.040
HbA1c, %		5.9 (5.7–6.3)	0.197	0.002
Hyperglycemia (>11 mmol/L)	No	219 (89%)	105.62 (83.05–127.42)	0.010
	Yes	26 (11%)	126.58 (94.85–171.39)	

LDL, low-density lipoprotein; HDL, high-density lipoprotein; LBP, lipopolysaccharide-binding protein.

Data are expressed as *n* (%), mean ± standard deviation (SD), or median (IQR). LPS is expressed as 3HM (pmol/L). *p*-values are given for the association between each variable and LPS plasma concentration. *p*-values presented are not corrected for multiple comparisons.

**Table 4:** Variables independently associated with LPS plasma concentration.

Variable	Standardized *β*	*β*	95% CI	*p*	VIF
Age, per year	0.192	0.835	0.347 to 1.322	0.001	1.179
BMI > 30 kg/m^2^	−0.018	−2.667	−20.081 to 14.747	0.763	1.162
HR, per log unit	0.053	29.479	−33.226 to 92.184	0.355	1.088
Diabetes	0.375	63.484	43.470 to 83.497	<0.001	1.196
TG, per g/L	0.235	11.427	5.653 to 17.201	<0.001	1.209
Total cholesterol, per g/L	0.204	26.337	10.73 to 41.944	0.001	1.246
Glycemia > 11 mmol/L	0.100	19.148	−3.246 to 41.542	0.093	1.179

*n* = 238 patients; *R*^2^ = 0.308; adjusted *R*^2^ = 0.287; Durbin–Watson test: 2.048; *F-*ratio = 14.650; *p* < 0.001. *β* values are regression coefficients.

### Relationship between endotoxemia and inflammation

Interleukin (IL)-6 and CRP were below the detection threshold in most patients (74% and 84%, respectively). We found no association between the measured cytokines [IL-6, IL-8, tumor necrosis factor (TNF)-α] and LPS (*p* > 0.05). A level of CRP higher than 10 mg/L was not associated with LPS (Table 5).

**Table 5:** Associations between LPS plasma concentration, inflammation, and short- and long-term outcomes.

Variable		*n* (%) or median (IQR) *N* = 245	Total LPS or *r*	*p*
Inflammation				
IL-6 > 3.2 (pg/ml)	No	181 (73.9%)	106.59 (83.69–131.00)	0.799
	Yes	64 (26.1%)	102.39 (82.00–132.47)	
IL-8 > 3.2 (pg/ml)	No	146 (59.6%)	107.98 (86.70–133.62)	0.144
	Yes	99 (40.4%)	104.51 (78.95–125.75)	
TNF-α (pg/ml)		9.31 (6.23–12.65)	−0.015	0.821
CRP > 10 mg/L	No	204 (83.6%)	106.50 (83.65–131.16)	0.617
	Yes	40 (16.4%)	103.72 (81.99–130.83)	
Short-term outcome (in-hospital)^a^				
Length of ICU stay, days		3 (3–4)	0.180	0.005
MACE	No	180 (73%)	108.37 (85.19–133.13)	0.119
	Yes	65 (27%)	97.48 (79.80–126.24)	
Death	No	242 (98.8%)	106.36 (83.37–130.84)	0.380
	Yes	3 (1.2%)	89.12 (52.89–x)	
Recurrent MI	No	236 (96.3%)	106.36 (83.65–131.58)	0.413
	Yes	9 (3.7%)	97.48 (68.46–127.93)	
Heart failure	No	187 (76.3%)	108.12 (83.62–132.69)	0.329
	Yes	58 (23.7%)	100.41 (81.23–127.18)	
Ventricular arrhythmia	No	230 (93.9%)	106.50 (83.72–131.71)	0.305
	Yes	15 (6.1%)	94.89 (82.22–124.62)	
Supraventricular arrhythmia	No	230 (93.9%)	106.25 (83.37–131.94)	0.676
	Yes	15 (6.1%)	110.44 (82.22–125.33)	
Long-term outcome (1-year)^b^				
MACE	No	212 (86.5%)	106.15 (83.09–130.58)	0.636
	Yes	33 (13.5%)	111.42 (86.28–132.56)	
Death	No	231 (94.3%)	106.32 (83.16–130.67)	0.941
	Yes	14 (5.7%)	108.65 (83.35–133.21)	
Recurrent MI	No	241 (98.4%)	106.18 (83.30–131.00)	0.859
	Yes	4 (1.6%)	112.45 (83.75–132.93)	
Heart failure	No	238 (97.1%)	106.25 (83.06–130.84)	0.399
	Yes	7 (2.9%)	116.15 (91.88–246.44)	
Unscheduled PCI	No	228 (93.1%)	106.15 (83.09–130.00)	0.364
	Yes	17 (6.9%)	113.90 (95.74–134.16)	
CABG	No	237 (96.7%)	106.40 (83.53–131.50)	0.400
	Yes	8 (3.3%)	99.89 (76.80–121.32)	
Unstable angina	No	244 (99.6%)	106.25 (83.23–131.16)	0.865
	Yes	1 (0.4%)	112.45	
Angina pectoris	No	243 (99.2%)	106.32 (83.44–130.67)	0.616
	Yes	2 (0.8%)	212.79 (80.26–x)	

LPS is expressed as 3HM (pmol/L). *p*-values are given for the association between each variable and LPS plasma concentration. *p*-values presented are not corrected for multiple comparisons.

^a^ In-hospital MACE: *CV death, recurrent MI, and HF.*

^b^ 1-year MACE: *CV death, recurrent MI, HF, unscheduled PCI, CABG, unstable angina, and angina pectoris.*

### Relationship between endotoxemia and short-term outcome

LPS blood concentration was not associated with initial severity (Table 2) nor with the occurrence of MACE (or any of its components) or all-cause mortality (*p* = 0.4) during hospital stay, as assessed by GRACE and SYNTAX scores. However, LPS was associated with a longer ICU stay (*r* = 0.18, *p* = 0.005) (Table 5).

### Relationship between endotoxemia and long-term (1-year) outcome

LPS blood concentration was not associated with the occurrence of 1-year MACE (or any of its components) nor with all-cause mortality (*p* = 0.9, Table 5).

## Discussion

Lipopolysaccharide plasma concentration at hospital admission was associated with metabolic conditions (in particular cholesterol, triglyceride concentrations, and diabetes) and age rather than with MI severity. LPS concentrations were not associated with inflammatory biomarkers, coronary artery disease severity assessed by angiography, or short-term and long-term occurrence of MACE.

When compared with measurements obtained using the same method and in the same laboratory, the LPS plasma concentrations in our cohort were slightly higher than those reported in healthy volunteers [106 (83–131) vs. 96 (77–116)] but lower than in patients with septic shock [106 (83–131) vs. 134 (126–142)] (20). These findings are in line with previous studies that reported increased endotoxin concentrations in patients with coronary artery disease (10, 21), which led to a hypothesis about the role of LPS proinflammatory activity in atherosclerosis development (22, 23) and MI pathogenesis (7). However, whether low-grade endotoxemia in MI patients is related to pre-existing metabolic conditions or acute digestive translocation linked to an MI event remains to be determined. Moreover, as LPS is mostly inactive in human blood, the LPS burden is imperfectly captured by LPS activity measurement (i.e., an increase in activity could represent either higher translocation or a decreased host inactivation capacity) (24). Our findings, which suggest an increased LPS mass concentration, further suggest an increase in gut-derived absorption rather than a default in the inactivation process.

Endotoxemia has been shown to produce low-grade inflammation, which is involved in the pathogenesis of obesity (25). Lipid and carbohydrate metabolism are also associated with endotoxemia (26). In particular, LPS absorption might occur through chylomicron metabolism, which is closely related to triglyceride absorption (27). Obesity is associated with dysregulations in lipid (i.e., hypertriglyceridemia, low HDL cholesterol, high LDL cholesterol) (28) and carbohydrate (i.e., hyperglycemia, hyperinsulinemia, and insulin resistance) (29) metabolism, both of which were associated with endotoxemia in our cohort. Nevertheless, there was no association between obesity and endotoxemia after adjustment for these confounding conditions. Therefore, our results give new insight into the relationship between obesity and endotoxemia by suggesting that this association is driven by metabolic alterations. During the acute phase of gut barrier disruption, PLTP negatively correlates with LPS concentration, probably reflecting increased elimination capacity (30–32). In contrast, the positive association between PLTP activity and LPS reported here might be related to the increase in PLTP activity documented in patients with metabolic syndrome (33), further suggesting the presence of metabolic endotoxemia in our cohort. We did not observe associations between endotoxemia and inflammation in our cohort, probably due to the early measurement of inflammatory biomarkers (before the peak was reached). We observed lower LPS levels in smokers. Whereas smoking and endotoxemia have been described as increasing the risk of incident atherosclerosis (23), previous studies did not report an association between smoking and increased endotoxin activity (34).

In a previous study reporting on endotoxemia attributed to gut barrier failure in the acute phase of MI, the authors noted a peak value for LPS activity on day 2 and associations between LPS concentration (delta day 2 − day 1) and adverse post-MI CV events (9). In our cohort, LPS measured at admission was associated with heart rate and shock index, suggesting a link between LPS and hemodynamic changes in MI. LPS is known to trigger inflammation, and these hemodynamic changes might reflect inflammation-related vasoplegia. LPS was also correlated to longer cardiac ICU length of stay, suggesting the clinical consequences of acute translocation. Nevertheless, we found no association between LPS measured at admission and post-MI adverse events, thus suggesting that LPS concentration measured at admission has few clinical consequences.

Some limitations need to be underlined. This was a single-center study; therefore, our findings require external validation. There was no sample size calculation, and some analyses might lack power. The inflammation parameters were taken very early (at hospital admission), and many measurements were below the threshold for detection (probably measured before peak). As a consequence, the relationships between LPS, vascular tone, and acute inflammation warrant further evaluation. Repeated or daily measurements of LPS could have provided additional information on the translocation mechanisms in the acute phase of MI. A combination of LPS activity and LPS mass measurement may also have provided further insight. A comparison with patients undergoing percutaneous coronary intervention without evidenced atherosclerosis and with atherosclerosis but no myocardial infarction could have provided additional information on the respective roles of metabolic and acute endotoxemia. Describing the reasons for patients’ ICU length of stay duration could have provided additional interesting information. We measured the total 3HM; thus, it is possible that part of the 3HM measured might be derived from human metabolites. However, bacterial LPS is the major part of total 3HM, and total 3HM is highly associated with LPS concentration (19).

In conclusion, at admission, higher LPS levels were related to metabolic alterations (cholesterol, triglyceride, and diabetes) and age. They were also associated with hemodynamic modifications (higher heart rate and shock ratio) and longer cardiac ICU length of stay but were not associated with inflammation and short- or long-term MACEs. Altogether, endotoxins measured at admission in patients with MI seemed to reflect low-grade metabolic endotoxemia due to pre-existing conditions leading to myocardial infarction rather than acute clinical severity. Endotoxins, therefore, do not appear to be a relevant therapeutic target for treating acute MI, but targeting endotoxemia could represent a promising strategy to prevent metabolic disorders and subsequent coronary artery disease progression. However, these findings require further confirmation.

## Data Availability

The raw data supporting the conclusions of this article will be made available by the authors without undue reservation.
